# Over a decade of experience in total knee arthroplasty with a multiradius design and fixed bearing at a single centre: Data from the Catalan Arthroplasty Registry

**DOI:** 10.1002/jeo2.12076

**Published:** 2024-07-02

**Authors:** Jordi Faig‐Martí, Adriana Martínez‐Catasús

**Affiliations:** ^1^ Orthopaedics Department Hospital Sant Rafael Barcelona Catalonia Spain

**Keywords:** arthroplasty registry, gender, multi‐radius knee, survival, total knee replacement

## Abstract

**Purpose:**

Arthroplasty registers can provide feedback information on the results of arthroplasties performed by a certain institution or surgeon. The use of real‐world data to achieve real‐world evidence can help evaluate the performance of the implants used and help counsel our patients. The main objective of our study was to determine the survivorship of the total knee implant we are currently using.

**Methods:**

A retrospective cohort study of patients who received a total knee arthroplasty from January 2009 to December 2020 in our hospital was conducted, using data from the Catalan arthroplasty registry and the Catalan health service database. Demographic and surgical data were analysed using the Kaplan–Meier method, log‐rank test and Cox proportional hazards models with the R Project software (*p* < 0.05).

**Results:**

A total of 1336 total knee arthroplasties were included in the study, of which 992 were women. The causes for revision included aseptic loosening (17), infection (29), instability (13), patellar implantation (13), arthrofibrosis (5) and quadriceps tendon rupture (1).

The cumulative risk for revision at 5 years using the Kaplan–Meier method was 6.0% and at 10 years 6.5%. Considering gender, this risk was 7.0% and 7.5% at 5 and 10 years, respectively, in women and 3.3% in men, both at 5 and 10 years (*p* = 0.009). A higher risk for revision in women was seen, which is considered statistically significant (*p* = 0.012).

**Conclusion:**

Our survivorship results are comparable to those published in the literature, but with a higher revision risk in women that is only statistically significant for the whole group of reoperations and for patellar implantation, but not for the rest of the diagnoses.

**Level of Evidence:**

Level IV.

**Abbreviations**
AOANJRR: Australian Orthopaedic Association National Joint Replacement Registry
CMBD‐ HA: minimum basic set of hospital discharge data
CR: Cruciate Retaining
PROM: patient‐reported outcome measures
PS: posterior‐stabilized
RACat: Catalan arthroplasty registry
RCA: central register of insured
RCT: randomized control trial
RWD: real‐world data
RWE: real‐world evidence
SKAR: Swedish Knee Arthroplasty Register
TKA: total knee arthroplasty

## INTRODUCTION

In the current context of continuous innovations and technological advances, the number of available prosthetic implants is increasing. In this scenario, it is crucial that medical professionals have access to scientific evidence on the models they use in surgery, in order to make decisions based on the principles of evidence‐based medicine and promote optimal patient outcomes.

Randomized control trials (RCTs) are often considered the best clinical study design. However, they are conducted including a limited number of patients who fulfil a series of requirements defined by the inclusion and exclusion criteria. This finally limits generalising the results of a certain RCT to the general population. This drawback is overcome by observational studies like those included in what is nowadays commonly referred to as real‐world evidence (RWE), to evaluate a certain treatment in routine clinical care. This idea, before it was given this name, kindled the creation of arthroplasty registries to monitor the results of joint implants in a general population [24].

Arthroplasty registries have shown that, while survivorship of a certain implant can show a general trend in different geographical settings, it differs from one country to another and even from one hospital to another in the same city [28]. The data compared from these registries are not those of an RCT with patients excluded according to the criteria defined in the study, but real‐world data (RWD) obtained prospectively from all kinds of patients with all kinds of comorbidities, life and exercise habits, diets, medications, height and weight. Furthermore, the institutions where the surgeries are performed are not all highly specialized orthopaedic centres where surgeons do a great volume of arthroplasties but all kinds of hospitals with different degrees of expertise.

Arthroplasty registers can provide feedback information on the results of arthroplasties performed by a certain institution or surgeon to encourage quality improvement [28, 29]. This can allow low‐performing institutions to seek excellence by improving their care processes in order to improve their quality indicators such as implant revision, readmission and complications.

The Catalan arthroplasty registry (RACat) was the first official arthroplasty registry in our country and was created in 2005 by the Catalan Society of Orthopaedic Surgery and Traumatology, the Catalan Health Service and the Catalan Agency for Health Information Assessment and Quality [1].

After almost 15 years now using the Genutech® total knee system (Surgival S.L.U.), without having previous published results on this implant, we wanted to evaluate our performance and compare the results with other studies based on the same or other registries in order to improve the care of our patients. This would allow us to have real‐world data to counsel our patients with real‐world evidence. A national arthroplasty registry reported a higher early revision for loosening and patellar pain in females [25]. The main aim of this study is to assess the survivorship of the total knee arthroplasty (TKA) we are currently using, with any type of revision as the primary endpoint, from a single independent centre. In addition, we analysed the different diagnoses for revision and the influence of gender on the revision rate.

## MATERIALS AND METHODS

### Study design

This is a single‐centre, retrospective cohort study with prospectively collected data, following the guidelines from the Strengthening the Reporting Observational Studies in Epidemiology (STROBE) statement [31].

### Data Sources

A single‐arm retrospective observational study using real‐world data [10] extracted from the Catalan arthroplasty registry and the Catalan health service database using the minimum basic set of hospital discharge data (CMBD‐HA) and Central Register of Insured (RCA) to obtain information on the patient status was undertaken. The bioethical committee from our hospital approved the study on 28 January 2021 with a waiver of informed patient consent because the study was a minimal‐risk database analysis with anonymized data.

From the RACat database, we selected the patients who had received a Genutech® total knee system (Surgival S.L.U.) during the period from January 2009 to December 2020. This is a complete system for cemented or hybrid knee arthroplasty with fixed bearing, with cruciate retaining (CR) or posterior‐stabilized (PS) options for stabilization, patellar option and multiradius design. In 2009, we started using this implant, and by 2020, we had a minimum 2‐year follow‐up. All patients who received a Genutech® total knee arthroplasty in our institution during this time period were included in the study. None of the patients in the database was excluded.

### Variables

We obtained demographic, surgical and implant information of every procedure, which was then completed with information regarding any revision, that is, any new surgical procedure the same knee joint could have sustained with the implantation of any component, obtained from the Catalan health service database. Unfortunately, our arthroplasty registry does not include information on postoperative complications if no new components were implanted or removed, nor does it include patient‐reported outcome measures (PROMs). Periarticular fractures were not included in this study.

The data extracted included demographic (age, gender, weight, body mass index and diagnosis), surgical (surgery time, time in ischaemia, surgical approach and intraoperative complications) and implant‐related information (stabilization, fixation and patellar resurfacing). Sex and gender are used in this report as synonyms since none of the patients included in the study declared any differences between their legal registered sex (understood as a biological classification) and their gender (considered the socially constructed roles of male, female or gender diverse).

### Data analysis

Demographic and surgical data were summarized as means or percentages as appropriate using standard statistics. Survival curves and estimates for 10‐year survival were created with the Kaplan–Meier method, and the log‐rank test was used to compare the differences between curves. Cox proportional hazards models were used to perform subgroup analyses, evaluating the following covariates: gender, tibial size, femoral size and type of stabilization. The R Project software (R Core Team 2022) was used for statistical analysis. The level of statistical significance was set at *p* < 0.05 (providing a 95% confidence interval).

### Surgical procedure

The usual procedure in our service for the patients studied in this series includes the use of a tourniquet during surgery, an articular drainage, nerve blocks for analgesia and ambulation after 24 h. Patients were discharged from the hospital after 4 to 5 days of the surgery and controlled at our clinic 10 to 14 days later. Radiological and clinical evaluation was performed at 1, 3, 6 and 12 months.

The implant used was the Genutech® total knee system that includes CR and PS options. The CR femoral component is available in noncemented (hydroxyapatite coated) and cemented versions. The femoral PS, tibial and patellar components are always cemented. The sizes available for tibia and femur range from 1 to 5 and polyethylene height ranges from 10 to 18 mm.

After 1 year of surgery, patients were controlled clinically and radiologically once a year until the fifth year. Patients were encouraged to come back for follow‐up every five years thereafter, but the patient's family doctor could refer the patient back to our institution if any problems with the knee arose.

## RESULTS

The database included 1336 Genutech® primary total knee arthroplasties performed in our hospital from 2009 to 2020 in 992 women and 344 men, with an average age of 73.27 (±7.07) and 72.75 (±7.98) years, respectively. From the total, 1049 were CR and 287 were PS. The patella was implanted in 225 cases (16.8%). In 379 cases, the femoral component was uncemented with hydroxyapatite coating, and all the tibial and patellar components were cemented. Preoperative diagnoses for the surgery included primary osteoarthritis (1266), secondary osteoarthritis (54), avascular necrosis [9] and rheumatic disease [7]. The mean follow‐up was 6.01 ( ± 3.12) years, with the longest follow‐up being 13.06 and the shortest 2 years.

At the end of the study period, 156 patients had died of unrelated causes, 18 patients were discharged due to the change of address and 78 cases (5.8%) had been revised, including 10 men and 68 women. The causes for revision included aseptic loosening [17], infection [29], instability [13], patellar implantation [13], arthrofibrosis [5] and quadriceps tendon rupture [1].

**Figure 1:**
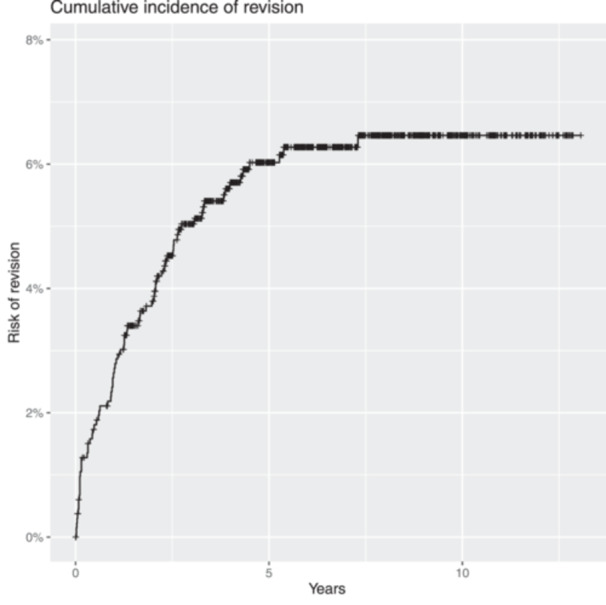
Cumulative incidence of revision.

The cumulative incidence of revision for any cause using the Kaplan–Meier method with a CI 95% was 6.0% (4.7%, 7.3%) at 5 years and 6.5% (5.0%, 7.9%) at 10 years, with at least 150 patients at risk at the beginning of this 10th year of follow‐up (Figure 1).

Considering only aseptic revisions except for secondary patellar implantation (aseptic loosening, instability arthrofibrosis and tendon rupture), the cumulative incidence of revision for the implant studied was 2.7% (1.8%, 3.7%) at 5 years and 3.2% (2.1%, 4.3%) at 10 years. On the other hand, the cumulative incidence of septic revisions was 2.3% (1.5%, 3.1%) at 5 years follow‐up and longer. Similarly, the cumulative incidence for patellar resurfacing posterior to the primary knee replacement was 1% (0.5%, 1.7%) after 5 years.

**Figure 2:**
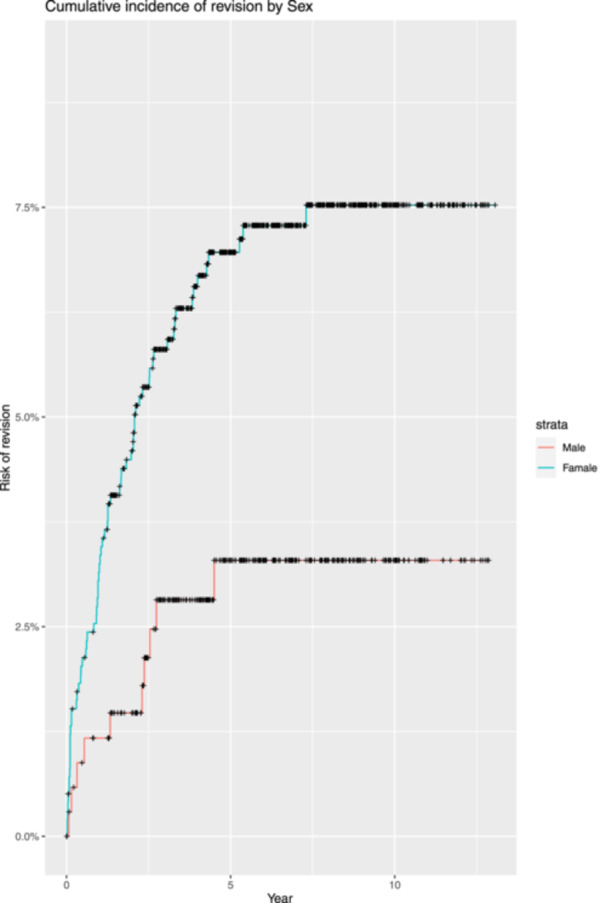
Cumulative incidence of revision by sex.

When considering gender, the cumulative incidence of revision was 7.0% (5.3%, 8.6%) and 7.5% (5.8%, 9.3%) at 5 and 10 years, respectively, in women, and 3.3% (1.2%, 5.3%) in men, both at 5 and 10 years. This difference is considered significant with a *p* value = 0.009 (Figure 2).

**Figure 3:**
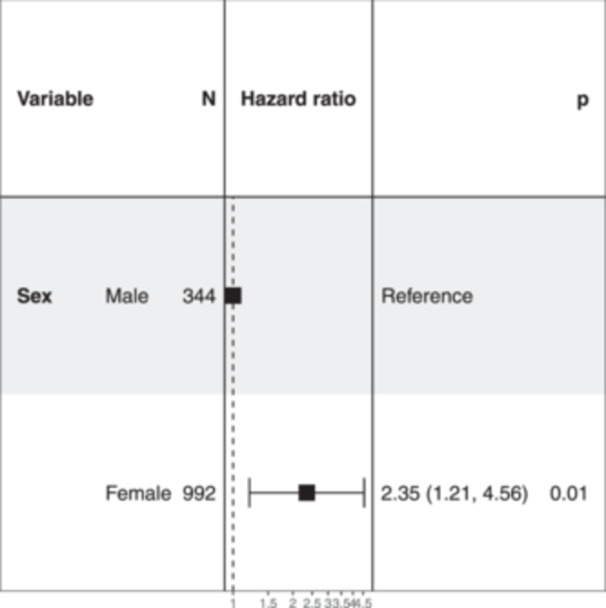
Cox proportional risk regression model by sex.

Using the Cox proportional risk regression model, a higher risk for revision in women is seen with a hazard ratio of 2.35 (CI 95%, 1.21–4.56) that is, considered statistically significant (*p* = 0.012) (Figure 3).

**Figure 4:**
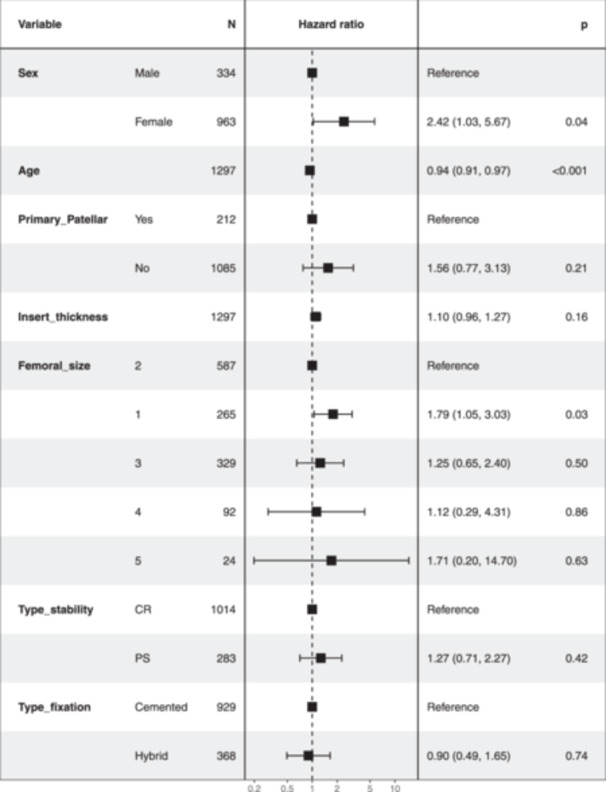
Cox proportional risk regression model with adjusting covariates sex, age, patellar resurfacing in primary knee replacement, insert thickness, femoral size, type of implant stability and type of cementation.

When adjusting the Cox model with other covariates that were also considered to be of interest (Figure 4), sex continued to appear as a significant factor (HR = 2.42; *p* = 0.04), as did age (HR = 0.94; *p* < 0.001) and femoral size (HR T1 = 1.79 over reference T2; *p* = 0.03). Although tibial size was of interest, it could not be fitted in the model because it did not meet the proportional hazards assumption for Cox regression.

To further evaluate the sex relationship with different diagnoses for revision, Fisher's exact test was performed showing a significative p‐value of 0.007 for reoperation in females in general and 0.027 for patellar resurfacing posterior to the primary knee replacement, but not statistically significative for the rest of the diagnoses that led to revision (Table 1).

**Table 1:** Cases and percentages of revision.

Characteristic	Female, *N* = 992^a^	Male, *N* = 344^a^	*p* Value^b^
Revision	68 (6.9%)	10 (2.9%)	0.007
Aseptic revision	30 (3.0%)	6 (1.7%)	0.2
Septic revision	25 (2.5%)	4 (1.2%)	0.14
Patellar revision	13 (1.3%)	0 (0%)	0.027*

^a^ *N*(%).

^b^ Pearson's *Χ* ^2^ test.

* Fisher's exact test.

The patella was replaced during primary knee replacement in 173 women and 40 men, and not replaced in 819 women and 304 men. Secondary patellar replacement for patellar pain was performed in 13 women (1%) and none in men.

## DISCUSSION

The results in our study show a higher revision rate compared to a previous report from RACat, which reported a risk of revision at 5 years (3.8%; 95% CI, 3.3–4.4) comparable to the other models in this registry (3.9%, 95% CI, 3.7‐4.1) [4]. This can be related to the fact that our institution would be a high‐volume hospital with 250 cases per year, and there is a higher incidence of revisions in those centres compared to low‐volume hospitals, as studies from the same registry show [2, 3]. In addition, other reports published by RACat [14] also suggest some association of an increased revision rate with the preference for no patellar resurfacing in primary TKA (6.79%; 95% CI, 6.45–7.14, at 15 years in bicompartmental TKA) versus increased patellar resurfacing (5.53%; 95% CI, 5.19–5.89, at 15 years in tricompartmental TKA).

Thus, our single‐centre study in a high‐volume hospital and a low preference for patellar resurfacing (24.9%) have allowed us to further analyse the reasons for the increased risk of revision due to these two factors. It therefore seems reasonable that aseptic revision risk is used to assess implant performance.

Arthroplasty registries and national patient registries provide similar information from a different perspective. While arthroplasty registries focus on the monitoring and analyses of a specific type of intervention (arthroplasty), national patient registries deal with vast numbers focused on public health care [27]. Linking information from both sources can boost our understanding of the performance of orthopaedic implants in a general population [15]. The analyses from arthroplasty data allow surgeons to monitor their results and spot underperforming implants [7, 26].

The survivorship of the implant studied is in line with that of other implants published and compares favourably with the results of large‐scale studies [12, 17]. Jorgensen, for example, reports 5973 aseptic revisions in a cohort of 478,081 primary total knee arthroplasties at 15 years follow‐up (3.0%) from the Australian Orthopaedic Association National Joint Replacement Registry (AOANJRR), with the tibial component being revised more commonly than the femoral one.

The trend towards a higher reoperation rate in females in our series is only statistically significant for the whole group of reoperations and for patellar implantation but not for the rest of the diagnoses. This can be due to the low number of reoperations that do not allow for a stratification in order to study each group of diagnoses separately. This trend, however, has already been noted in the literature and merits further studies [9]. The 2020 Annual Report of the Swedish Knee Arthroplasty Register (SKAR) [27, p. 25] reflects the difficulty of evaluating the effect of sex on the risk of revision and reports a higher early revision for infection in males, but higher early revision for loosening and patellar pain in females, without being able to explain these differences. The Australian Registry [5] also reports a higher revision rate in males, increased with lower age, especially related to infection (pp. 212–213). They also report a higher revision rate in younger females.

Increased generalized ligament laxity or the difference in muscular strength could play a role in the differences observed in reoperation rates seen after total knee replacements [18]. Sex differences in the perception of pain [23] and a higher rate and severity of metal sensitization in females [8] could also account for these differences in revision rates.

Some studies have shown an increase in reoperations in young male patients [6, 12, 17]. In our study, the average age of patients was much higher than that of the studies mentioned and only four patients were under 50 years of age. However, our results show that increasing age at total knee replacement is shown to be a protective factor, as younger patients have a higher risk of revision. Jorgensen [17] reports 7.8% revisions in patients under 55 years of age, as opposed to 1.0% in patients older than 75.

A different approach to diagnosis and treatment depending on the patient's gender was suggested some years ago [21]. Anatomical differences between the male and female knees led to the development of gender‐specific implants, which were later proven not to be necessary [16], because no differences in outcome were observed between patients with a gender‐specific knee arthroplasty and a unisex arthroplasty. However, differences do exist between postoperative function scores and activity levels between males and females [11]. Furthermore, worse postoperative outcomes have been related to more advanced osteoarthritis at the time of surgery, which is the case in female patients [13].

Considering the eternal dilemma concerning patella replacement, the Australian Registry [5] reports as high as 76.1% of patellar resurfacing in 2021 (p. 199), which decreases to 2.8% in Sweden in 2019 [25, p. 32], with the rate in our study being in between with 16.8%, which is slightly higher than the rate reported by the Finnish registry [20]. In our series, primary patellar replacement depended on the surgeon's decision. One of them routinely replaced the patella while the other five hardly ever did. Our data show a reoperation rate for those cases without a primary replacement of 1% in women and none in men. With this low reoperation rate and the potential complications of patellar implantation, the pros and cons of primary patellar replacement should be balanced. The SKAR reports a higher percentage of patellar replacement in women, attributed to patella‐femoral pain being more frequent in women. The routine resurfacing of the patella would, of course, reduce this reoperation rate [19, 30] but possible complications of this practice such as extensor mechanism rupture, mal‐tracking, patella polyethylene wear, patella fracture and fragmentation [22] have to be taken into account. Our current practice is to replace the patella only in those cases where there are clear symptoms of patellofemoral pain.

### Strengths and limitations

The accuracy and completeness of data supplied to a database are arguably the main limitations of studies performed with it. In our case, the Catalan arthroplasty registry has been improving the quality of its data due to policies that link funding of hospitals in the national health service with the supply of information regarding certain procedures. However, we were able to improve the quality and quantity of data available by linking two databases (RACat and the Catalan health service database) [15].

As the need for a component revision was the only outcome we were able to assess, as is usually the case in registry‐based studies, we have no information regarding patient satisfaction and other patient‐reported outcomes or radiological data of nonsymptomatic radiological loosening.

Management of the patella in the primary operation was different among the surgeons in the team, which might represent a bias in the results regarding reoperations for patellar pain.

The high survival rate of the implant studied reduces the data available on risk factors for revisions. The number of reoperations in this study is too low to allow for a stratification of diagnoses that would allow further insight into risk factors for reoperation.

Finally, the fact that the same team performed the surgeries, and postoperative care was undertaken by the same nursing and physiotherapy professionals, represents a homogeneous management of the group of patients included in this study.

## CONCLUSIONS/OVERVIEW

The data presented in this study summarize the results that can be obtained in a real‐world setting using a particular model of total knee replacement. Better results regarding revision surgery could probably be obtained by selecting patients included in the study or selecting surgeons that perform only total knee replacements instead of General Orthopaedics. However, as stated in the introduction, our goal was to report on RWD.

Total knee replacement with the implant studied shows an acceptable percentage of revisions for aseptic complications at 10 years of follow‐up, with septic complications being of similar relevance up to 5 years. Female patients without a primary patellar implantation required secondary surgery for patellar pain much more frequently than male patients. Future work will have to focus on elucidating the cause of this gender difference in the evolution of total knee replacements and on determining the best benefit–risk ratio for patellar interventions, whether primary knee replacement or secondary patellar intervention.

## AUTHOR CONTRIBUTIONS

All authors contributed to the study's conception and design. Material preparation, data collection and analysis were performed by Jordi Faig‐Martí. The first draft of the manuscript was written by Jordi Faig‐Martí and Adriana Martínez‐Catasús, and all authors commented on previous versions of the manuscript. All authors read and approved the final manuscript.

## CONFLICT OF INTEREST STATEMENT

The authors declare no conflicts of interest.

## ETHICS STATEMENT

Ethical approval was waived by the Ethics Committee of our Institution in view of the retrospective nature of the study and all the procedures being performed were part of the routine care (approval reference PR‐2021‐01).

## Data Availability

The data that support the findings of this study are available on request from the corresponding author. The data are not publicly available due to privacy or ethical restrictions.
